# Soil chemistry, metabarcoding, and metabolome analyses reveal that a sugarcane—*Dictyophora indusiata* intercropping system can enhance soil health by reducing soil nitrogen loss

**DOI:** 10.3389/fmicb.2023.1193990

**Published:** 2023-05-25

**Authors:** Mingzheng Duan, Yijie Li, Guanghu Zhu, Xiaojian Wu, Hairong Huang, Jie Qin, Shengfeng Long, Xiang Li, Bin Feng, Sunqian Qin, Qi-Huai Liu, Changning Li, Lingqiang Wang, Qing Li, Tieguang He, Zeping Wang

**Affiliations:** ^1^Sugarcane Research Institute, Guangxi Academy of Agricultural Sciences/Sugarcane Research Center, Chinese Academy of Agricultural Science/Key Laboratory of Sugarcane Biotechnology and Genetic Improvement (Guangxi), Ministry of Agriculture, Nanning, China; ^2^Key Laboratory for Conservation and Utilization of Subtropical Agro-Bioresources, College of Agriculture, Guangxi University, Nanning, China; ^3^Center for Applied Mathematics of Guangxi (GUET), Guilin, China; ^4^Guangxi Academy of Agricultural Sciences, Nanning, China; ^5^Laibin Academy of Agricultural Sciences, Laibin, China

**Keywords:** intercropping system, NPK, soil ions, soil enzyme activity, metabarcoding, soil metabolome

## Abstract

**Introduction:**

Greater amounts of fertilizer are applied every year to meet the growing demand for food. Sugarcane is one of the important food sources for human beings.

**Methods:**

Here, we evaluated the effects of a sugarcane—*Dictyophora indusiata* (DI) intercropping system on soil health by conducting an experiment with three different treatments: (1) bagasse application (BAS process), (2) bagasse + DI (DIS process), and (3) the control (CK). We then analyzed soil chemistry, the diversity of soil bacteria and fungi, and the composition of metabolites to clarify the mechanism underlying the effects of this intercropping system on soil properties.

**Results and discussion:**

Soil chemistry analyses revealed that the content of several soil nutrients such as nitrogen (N) and phosphorus (P) was higher in the BAS process than in the CK. In the DIS process, a large amount of soil P was consumed by DI. At the same time, the urease activity was inhibited, thus slowing down the loss of soil in the DI process, while the activity of other enzymes such as β-glucosidase and laccase was increased. It was also noticed that the content of lanthanum and calcium was higher in the BAS process than in the other treatments, and DI did not significantly alter the concentrations of these soil metal ions. Bacterial diversity was higher in the BAS process than in the other treatments, and fungal diversity was lower in the DIS process than in the other treatments. The soil metabolome analysis revealed that the abundance of carbohydrate metabolites was significantly lower in the BAS process than in the CK and the DIS process. The abundance of D(+)-talose was correlated with the content of soil nutrients. Path analysis revealed that the content of soil nutrients in the DIS process was mainly affected by fungi, bacteria, the soil metabolome, and soil enzyme activity. Our findings indicate that the sugarcane–DIS intercropping system can enhance soil health.

## 1. Introduction

The sustainable production of food has long been an important goal for mankind, and this is being increasingly challenged by the unabated growth of the human population and the intensification of climate change (Zhang et al., 2018; Singh et al., 2020). Large quantities of fertilizer have been applied to accommodate the growing demand for food. However, the excessive application of fertilizer has had deleterious effects both on crop production (e.g., decreased yields) and the environment (e.g., environmental pollution, reductions in soil biodiversity) in both China and several other developing countries (Vitousek et al., 2009; Li et al., 2019; Cui et al., 2020; Xu et al., 2020). There is thus a pressing need to develop approaches that provide some of the beneficial effects of fertilizer applications without compromising agricultural productivity and soil environments (Duan et al., 2022). Sugarcane (*Saccharum* spp.) is the world’s largest sugar crop, which widely used in food and industrial fields (Singh et al., 2020). Bagasse is a plant residue that is generated following the extraction of sugar from crops such as sugarcane. It is known that inorganic nutrients (e.g., phosphorus (P) and potassium (K)) required for the growth of plants are abundant in bagasse (Sharma et al., 2015). Bagasse is thus often used as an auxiliary fertilizer in sugarcane fields after harvest and sugar extraction (Seleiman and Kheir, 2018). The application of bagasse is considered an effective strategy for alleviating the deleterious effects of excessive fertilizer application and more generally for achieving the goals of ecological agriculture, which aims to enhance production while promoting ecological sustainability. Bagasse application has been shown to enhance the health of soil by increasing the abundance of various soil nutrients (Sharma et al., 2015; Seleiman and Kheir, 2018; Bento et al., 2019) and pH (Mohamed et al., 2019). Bagasse application also promotes the activity of soil microbes and increases the diversity of soil microbial communities (Bhadha et al., 2020; Ullah et al., 2020).

*Dictyophora indusiata* (DI), which is in the family Phallaceae, is an economically valuable, edible mushroom that is used in Chinese cuisine (Duan et al., 2023). DI is also widely used as a Chinese medicine material (Wang et al., 2021). Some agricultural waste including bamboo and sugarcane bagasse can be used to grow DI and improve soil nutrients (Tong et al., 2021; Thaisuchat et al., 2022). Consequently, the application of bagasse to sugarcane fields can enhance sugarcane production and promote soil health. However, whether bagasse application can have positive effects on the cultivation of DI when it is intercropped with sugarcane remains unclear. Therefore, additional research is needed to explore approaches that could be employed to enhance the abundance of soil nutrients in sugarcane–DI intercropping systems.

Soil health is affected by various biotic and abiotic factors, and microbes (especially bacteria and fungi) are one of the most important factors affecting soil biogeochemical cycles and the transformation of soil nutrients (Basu et al., 2021). Soil enzymes are mainly derived from soil microbes, and concentrations of soil nutrients are substantially affected by soil microbial activities. One previous study has shown that urease activity can have a major effect on the release of nitrogen (N) in soil (Cantarella et al., 2018). In sugarcane–DI intercropping systems, DI is the main fungus mediating the transformation of soil; this has major implications for soil health because the presence of a single, dominant fungus in the soil can alter the composition of metal ions in soil and thus affect the absorption of metal ions by other organisms (Zotti et al., 2021; Duan et al., 2022). Soil metabolomics can be used to clarify correlations of metabolic fingerprints with environmental factors, soil nutrients, microbial diversity, and plant phenotypes (Withers et al., 2020). For example, changes in the abundance of soil carbohydrate metabolites can be affected by the content of soil organic matter and microbial activity (Vranova et al., 2013; Gunina and Kuzyakov, 2015). This approach can thus be used to identify key metabolite markers associated with soil health.

Here, we evaluated the effects of a sugarcane–DI intercropping system on soil health through a field experiment. We then conducted analyses of soil chemistry, soil fungi and bacteria diversity, as well as the metabolite composition to clarify the mechanisms by which the sugarcane–DI intercropping system enhanced soil health. Our study suggests a new cultivation model to preserve soil fertility and increase economic value of sugarcane fields.

## 2. Materials and methods

### 2.1. Materials

**Figure 1:**
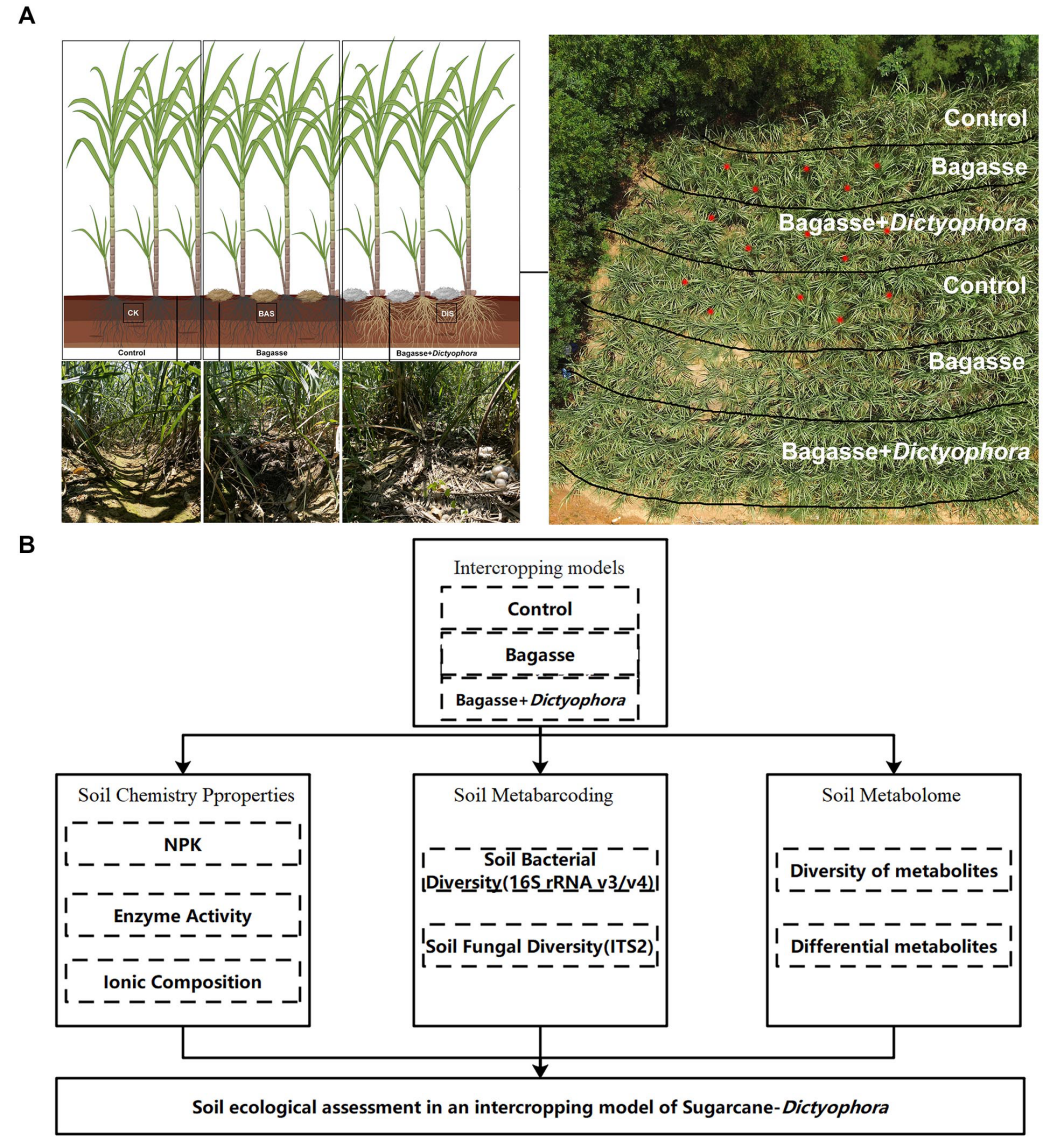
The experimental approach used in our study to characterize the effects of the sugarcane–DI intercropping system on soil health. **(A)** Experimental design with three treatments. The three images in the lower left show sugarcane rows corresponding to the different treatments. The image in the upper right shows an aerial view of the experimental field and the division of the treatments within the experimental field; soil samples were taken at each of the red dots shown in this image. **(B)** Schematic diagram of the analytical procedures used to evaluate the effects of each treatment on soil properties. The Figure 1A were build in www.biorender.com.

The experiment was conducted in a field located in Laibin City, Guangxi, China (23°16′N, 108°24′E) from February to August 2022. There were three treatments during the experiment: control (CK), in which sugarcane was grown under normal management conditions; BAS process, in which crushed, air-dried bagasse was applied to the soil between rows of sugarcane plants; and DIS process, in which crushed, air-dried BAS, mixed with DI strains obtained via wheat grain fermentation, was applied to the soil between rows of sugarcane plants (Figure 1A). The sugarcane variety GT42 was obtained locally and was used in all experiments. Sugarcane plants were grown with approximatively a 50 cm interrow distance, and the depth of each row was 30 cm of each ridge. When the height of sugarcane plants reached approximately 50 cm after 60 days of cultivation, we demarcated five semi-rectangular areas in which the three treatments were performed. Two duplicates of the CK and BAS process were performed, and the DIS process was only performed in one of these areas, which was located in the middle of the experimental site (Figure 1A). Fruiting bodies of DI matured after approximately 90 d, and high soil moisture levels were maintained during this period. Five soil samples were taken from the three processes (CK, BAS, and DIS), respectively, in the experimental area (red dots in Figure 1A).

After the surface bagasse was cleaned, soil samples were collected from the topsoil layer at a depth of 0–10 cm. The soil was then sieved through a 0.425 mm filter, packed into sterile cryostorage tubes, and frozen in liquid nitrogen.

### 2.2. Methods

Various analytical methods shown in Figure 1B were used to evaluate the effect of the sugarcane–DI intercropping system on soil properties.

#### 2.2.1. Soil chemical properties

The Kjeldahl method with sulfuric acid–accelerator digestion was used to measure the content of total N and basic nitrogen (BN) in soil (Jiao et al., 2015). NaOH alkali melting and molybdenum–antimony resistance spectrophotometry were used to measure the content of total P in soil (Xiao et al.,1988). NaOH alkali melting and flame photometry were used to measure the content of total K in soil (Fu et al., 1988). A carbon and N analyzer was used to measure the content of soil organic carbon (OC) (Dalian Environmental Monitoring Center, 2014). Molybdenum–antimony resistance colorimetry with ammonium fluorine-hydrochloric acid solution and sodium bicarbonate solution was used to measure the content of available P (AP) (Soil Testing-Part7, 2014). Ammonium acetate extraction and flame photometry were used to measure the content of available K (AK) in soil (Du et al., 2004). A Micro Soil Urease Assay Kit (BC0125-100 T/48S, Solarbio, Beijing, China) was used to determine the activity of soil urease. The 3, 5-dinitrosalicylic acid colorimetric method was used to measure the content of sucrase in soil [30; 31]. The *p*-nitrophenol colorimetric method was used to determine the content of β-glucosidase (Eivazi and Tabatabai, 1988). The 3, 5-dinitrosalicylic acid colorimetric method was used to determine the content of cellulase in soil (Li et al., 2008; Lin, 2010). An Assay Kit (YX-C-B913, Lai Er Bio-Tech, Hefei, China) was used to determine the laccase activity in soil. An Assay Kit (YX-C-B625, Lai Er Bio-Tech, Hefei, China) was used to determine the amylase activity in soil. After deionized water extraction, inductively coupled plasma optical emission spectroscopy (ICP-OES) was used to measure the content of 27 ions in soil (Bao, 2000). IBM SPSS Statistics 26.0 software was used to compute variances in each of the soil chemical properties.

#### 2.2.2. Metabarcoding analysis

A HiPure Soil DNA Kit (Magen, Guangzhou, China) was used to extract DNA from soil samples (3 g) following the manufacturer’s instructions according to a previously described method (Duan et al., 2022). Polymerase chain reaction (PCR) of the 16S rDNA V3–V4 region in the ribosomal RNA gene was conducted using the primers 341F (5′-CCTACGGGNGGCWGCAG-3′) and 806R (5′-GGACTACHVGGGTATCTAAT-3′) for bacteria (Guo et al., 2017) and the primers ITS3_KYO2 (5′-GATGAAGAACGYAGYRAA-3′) and ITS4 (5′-TCCTCCGCTTATTGATATGC-3′) for fungi (Toju et al., 2012). An Illumina NovaSeq 6,000 sequencing platform was used to paired-end sequence the purified amplicons, which were pooled in equimolar ratios following the standard protocol. The clean tags were clustered into operational taxonomic units (OTUs) of ≥97% similarity using UPARSE (Edgar, 2013) (version 9.2.64) pipeline. The tag sequence with highest abundance was selected as representative sequence within each cluster. A naïve Bayesian model using the Ribosomal Database Project classifier (version 2.2) (Wang et al., 2007) based on SILVA (16S rRNA OTUs) database (Pruesse et al., 2007) (version138.1) and UNITE (ITS OTUs) database (Nilsson et al., 2019) (version 8.3) with the confidence threshold value of 0.8, which is a naïve Bayesian classifier, was used to classify the representative operational taxonomic unit (OTU) sequences. R software was used to create all figures. The VennDiagram package (version 1.6.16) in R software was used to compare OTUs among groups (Chen and Boutros, 2011). Principal component analysis (PCA) and Shannon index analysis were performed to identify the between-group variance. QIIME (version 1.9.1) software was used for Shannon, Chao1, ACE, Simpson and Good’s coverage index calculations (Caporaso et al., 2010). For PCA analysis, first, OTUs numbers were normalized into log_2_, then vegan package (version 2.5.3; http://CRAN.R-project.org/package=vegan; 2022.11.9) in R software was used to conduct PCA to clarify variation in the composition of OTUs among experimental groups.

#### 2.2.3. Soil metabolome analysis

Soil samples (0.5 g) were mixed with 1 mL of a methanol:isopropanol:water (3:3:2 v/v/v) solution, vortexed for 3 min, and placed on an ultrasound which performed in ice water for 20 min. After centrifugation of the extract at 12,000 r/min at 4°C for 3 min, the supernatant was mixed with 0.020 mL of internal standard (10 μg/mL) in a sample vial, followed by evaporation under N flow. A lyophilizer was used to freeze-dry the sample, and the residue was used for derivatization. The sample was then incubated at 37°C for 2 h after being mixed with 0.1 mL of methoxyamine hydrochloride in pyridine (0.015 g/mL). Next, 0.1 mL of bis-(trimethylsilyl)-trifluoroacetamide with 1% trimethylchlorosilane was added to the mixture and maintained at 37°C for 30 min after vortex mixing. After mixing approximately 0.2 mL of the derivatization solution with n-hexane and diluting to 1 mL, the mixture was filtered through a 0.22 μm organic phase syringe filter and placed in a refrigerator at-20°C; the mixture was then analyzed within 24 h. Gas chromatography–mass spectrometry was conducted on an Agilent 8,890 gas chromatograph with a 5977B mass spectrometer and a DB-5MS column (30 m length × 0.25 mm i.d. × 0.25 μm film thickness, J&W Scientific, United States) to analyze the metabolites in soil samples. The carrier gas used was helium, and the gas flow rate through the column was 1.2 mL/min. Injections of 1 μL were conducted in front inlet mode at a split ratio of 5:1. The oven temperature was maintained at 40°C for 1 min and then increased to 100°C at 20°C/min; it was then increased to 300°C at 15°C/min and held at 300°C for 5 min. The scan mode was used in analyses of all samples. The ion source temperature and transfer line temperature were 230°C and 280°C, respectively. Metware’s metabolite database (v2.0; Metware Biotechnology Co., Ltd. Wuhan, China), as well as publicly available databases, including MassBank (http://www.massbank.jp (accessed on 20 October 2022), HMDB (Human Metabolome Database; http://www.hmdb.ca (accessed on 20 October 2022)), and METLIN (http://metlin.scripps.edu/index.php (accessed on 20 October 2022)), were used to qualitatively analyze primary and secondary mass spectrometry data. Unsupervised PCA was conducted using the function prcomp in R (www.r-project.org), and all data were unit variance-scaled prior to the unsupervised PCA.

#### 2.2.4. Correlation analysis

SmartPLS4 software^1^ was used to construct a partial least squares path model (PLS–PM). Cronbach alpha index values for each latent variable in the PMs were greater than 0.3, and *p*-values were evaluated using 20,000 bootstrap replicates. OmicShare Tools (https://www.omicshare.com/tools (accessed on 20 November 2022)) was used to conduct canonical correlation analysis (CCA). All data were log_2_-transformed prior to analyses.

## 3. Results

### 3.1. Soil chemistry properties

#### 3.1.1. The BAS and DIS process had a significant effect on the content of P, OC, AP, and AK

First, the main soil nutrient indicators, including N, P, K, pH, OC, AP, BN, and AK, in soil from the surface layer among the three experimental groups (CK, BAS process, and DIS process) were compared. The BAS process and DIS process had a significant effect on the content of P, OC, AP, and AK (*F*_2,12_ > 100) (Table 1). Soil P content (CK: 0.66 g/kg; BAS: 0.79 kg) and AP (69.02 mg/kg, 142.46 mg/kg) were higher in the BAS process (0.79 g/kg and 142.46 mg/kg, respectively) than in the CK (0.66 g/kg and 69.02 mg/kg, respectively), while OC content was lower in the BAS process (11.54 mg/kg) than in the CK (15.62 mg/kg). Moreover, P, AP, and AK contents were lower in the DIS process (0.59 g/kg, 65.7 mg/kg, and 92.4 mg/kg, respectively) than in the BAS process (0.79 g/kg, 142.76 mg/kg, and 125.4 mg/kg). It was also noticed that soil pH was higher in the BAS process (4.84) than in the CK (4.26), and soil pH was lower in the DIS process (4.17) than in the CK. Although differences were observed in the content of other nutrients among treatments, such as the content of N, K, and BN, differences in the content of these nutrients among treatments were not significant.

**Table 1:** Different soil nutrients content of soil samples in the BAS process and DIS process.

ID	CK	BAS	DIS	*F*-value	CK/BAS	BAS/DIS
N(g/kg)	1.45	1.57	1.503	8.449	↑**	ns
P(g/kg)	0.66	0.79	0.59	153.415	↑***	↓***
K(g/kg)	5.22	4.81	4.99	11.164	↓**	ns
pH	4.26	4.84	4.17	37.73	↑***	↓***
OC(mg/kg)	15.62	11.54	15.04	511.063	↓***	↑***
AP(mg/kg)	69.02	142.46	65.7	1781.229	↑***	↓***
BN(mg/kg)	153.42	163.34	142.42	82.226	↑***	↓***
AK(mg/kg)	120.8	125.4	92.4	3,686	↑***	↓***

Numbers are the means of five samples; “/” indicates versus; “↑ and ↓” indicate up-regulated and down-regulated, respectively; OC, organic carbon; AP, available phosphorus; BN, basic nitrogen; AK, available potassium; “*” indicates *p*-value between 0.01 and 0.05; “**” indicates *p* between 0.001 and 0.005; “***” indicates *p*-value less than 0.001; “ns” indicates not significant.

#### 3.1.2. The DIS process restrains soil urease activity

We measured the activity of six soil enzymes, urease, sucrase, β-glucosidase, cellulase, laccase, and amylase, that play a key role in shaping soil chemical properties. The BAS process and the DIS process had significant effects on the activities of all enzymes according to analysis of variance (*F*_2,12_ > 100) (Table 2). The activity of cellulase in soil was higher in the BAS process (1.19 U/g) than in the CK (0.91 U/g); the activity of urease, sucrase, and β-glucosidase was lower in the BAS process (1,228.95 U/g, 3.28 U/g, and 12. 01 U/g, respectively) than in the CK (1,326.66 U/g, 9.87 U/g, and 19.35 U/g, respectively). The activity of sucrase, β-glucosidase, cellulase, laccase, and amylase was higher in the DIS process (9.23 U/g, 31.17 U/g, 1.68 U/g, 9.92 U/g, and 1.45 U/g, respectively) than in the BAS process (3.28 U/g, 12.01 U/g, 1.19 U/g, 1.51 U/g, and 0.87 U/g, respectively). Urease activity in soil was lower in the DIS process (867.52 U/g) than in the BAS process (1,228.95 U/g); the activity of urease was higher than that of all five other enzymes examined. These findings indicate that the BAS process and the DIS process can have significant effects on the activity of soil enzymes.

**Table 2:** Soil enzymes activity in the BAS process and the DIS process.

ID	CK	BAS	DIS	*F*-value	CK/BAS	BAS/DIS
urease	1326.66	1228.95	867.52	125.06	↓**	↓***
sucrase	9.87	3.28	9.23	830.01	↓***	↑***
β-glucosidase	19.35	12.01	31.17	345.86	↓***	↑***
cellulase	0.91	1.19	1.68	112.32	↑***	↑***
laccase	1.18	1.51	9.92	1185.56	ns	↑***
amylase	0.67	0.87	1.45	51.89	ns	↑***

The numbers are means of five soil samples. The units are U/g; “/” indicates versus; “↑ and ↓” indicate up-regulated and down-regulated, respectively; “*” indicates *p*-value between 0.01 and 0.05; “**” indicates *p*-value between 0.001 and 0.005; “***” indicates *p*-value less than 0.001; “ns” indicates not significant.

#### 3.1.3. The content of soil ions less affected by both BAS and DIS processes

We measured the content of common ions in soil using ICP–OES to clarify the effects of the BAS process and the DIS process on the composition of ions in soil. The mean content of zinc (Zn), iron (Fe), K, titanium (Ti), magnesium (Mg), calcium (Ca), P, and sodium (Na) in soil was greater than 1 mg/L, indicating that these ions were major components of the soil; the mean content of barium (Ba), manganese (Mn), cerium (Ce), vanadium (V), rubidium (Rb), chromium (Cr), lanthanum (La), strontium (Sr), (lead) Pb, lithium (Li), nickel (Ni), thorium (Th), niobium (Nb), scandium (Sc), copper (Cu), gallium (Ga), cobalt (Co), beryllium (Be), and molybdenum (Mo) was less than 1 mg/L, indicating that these ions were minor components of the soil (Table 3). We characterized variation in the content of 27 ions in soil between treatments, and the average *F*-value for the 27 ions was 32. The content of ions in soil was not greatly affected by the BAS process and the DIS process, as significant differences in the content of most ions between the BAS process and the DIS process were not observed (e.g., Zn content) and variation among treatments was low (e.g., *F*-value in the Zn content was less than 32). However, the *F*-values of six ions of interest (Fe, Ca, P, Cr, La, and Mo) were greater than 32 (33, 300.345, 93.091, 37, 41, and 127, respectively). The content of Ca and La was higher in the BAS process than in the CK; the content of Fe, Cr, and Mo was lower in the BAS process than in the CK. The content of P and Mo was lower in the DIS process than in the CK. Data on the content of the above ions converted into unit soil weight (mg/kg) are shown in Supplementary Table S1.

**Table 3:** Soil metal ions content in the BAS process and DIS process.

ID	CK	BAS	DIS	*F*-value	CK/BAS	BAS/DIS
Zn(mg/L)	189.11	163.59	162.96	7.546	↓**	ns
Fe(mg/L)	102.82	83.31	83.4	33.571	↓***	ns
K(mg/L)	24.32	21.42	20.56	16.176	↓**	ns
Ti(mg/L)	11.68	10.73	10.54	5.33	↓*	ns
Mg(mg/L)	10.04	9.28	9.69	3.118	↓*	ns
Ca(mg/L)	1.46	3.35	1.93	300.345	↑***	↓***
P(mg/L)	1.58	1.66	0.99	93.091	ns	↓***
Na(mg/L)	1.18	1.02	0.88	16.781	↓*	↓*
Ba(ug/L)	376.1	359.86	349.13	6.092	ns	ns
Mn(mg/L)	0.33	0.34	0.34	0.411	ns	ns
Ce(ug/L)	321.71	320.48	337.49	1.055	ns	ns
V(ug/L)	269.6	229.75	224.57	24.751	↓***	ns
Rb(ug/L)	213.79	173.35	186.98	22.624	↓***	↑*
Cr(ug/L)	192.72	160.69	153.46	37.273	↓***	ns
La(ug/L)	127.27	158.31	164.04	40.912	↑***	ns
Sr(ug/L)	117.02	112.44	119.68	1.067	ns	ns
Pb(ug/L)	94.81	79.35	80.01	11.788	↓**	ns
Li(ug/L)	84.66	68.88	68.69	16.266	↓***	ns
Ni(ug/L)	68.14	53.38	55.12	25.241	↓***	ns
Th(ug/L)	53.94	59.43	59.79	12.44	↑**	ns
Nb(ug/L)	55.44	52.41	51.35	2.049	ns	ns
Sc(ug/L)	45.75	48.33	46.46	2.075	ns	ns
Cu(ug/L)	48.6	44.43	42.83	6.703	ns	ns
Ga(ug/L)	44.92	39.27	38.11	21.784	↓***	ns
Co(ug/L)	27.96	22.29	21.94	20.265	↓***	ns
Be(ug/L)	4.53	3.77	3.56	14.214	↓**	ns
Mo(ug/L)	0.99	0.87	0.68	126.819	↓***	↓***

The number indicates the mean value of five soil samples; “/” indicates versus; “↑ and ↓” indicate up-regulated and down-regulated, respectively; “*” indicates *p*-value between 0.01 and 0.05; “**” indicates *p*-value between 0.001 and 0.005; “***” indicates *p*-value less than 0.001; “ns” indicates not significant.

### 3.2. The diversity of soil fungi and bacteria inversely impacted by BAS and DIS

**Figure 2:**
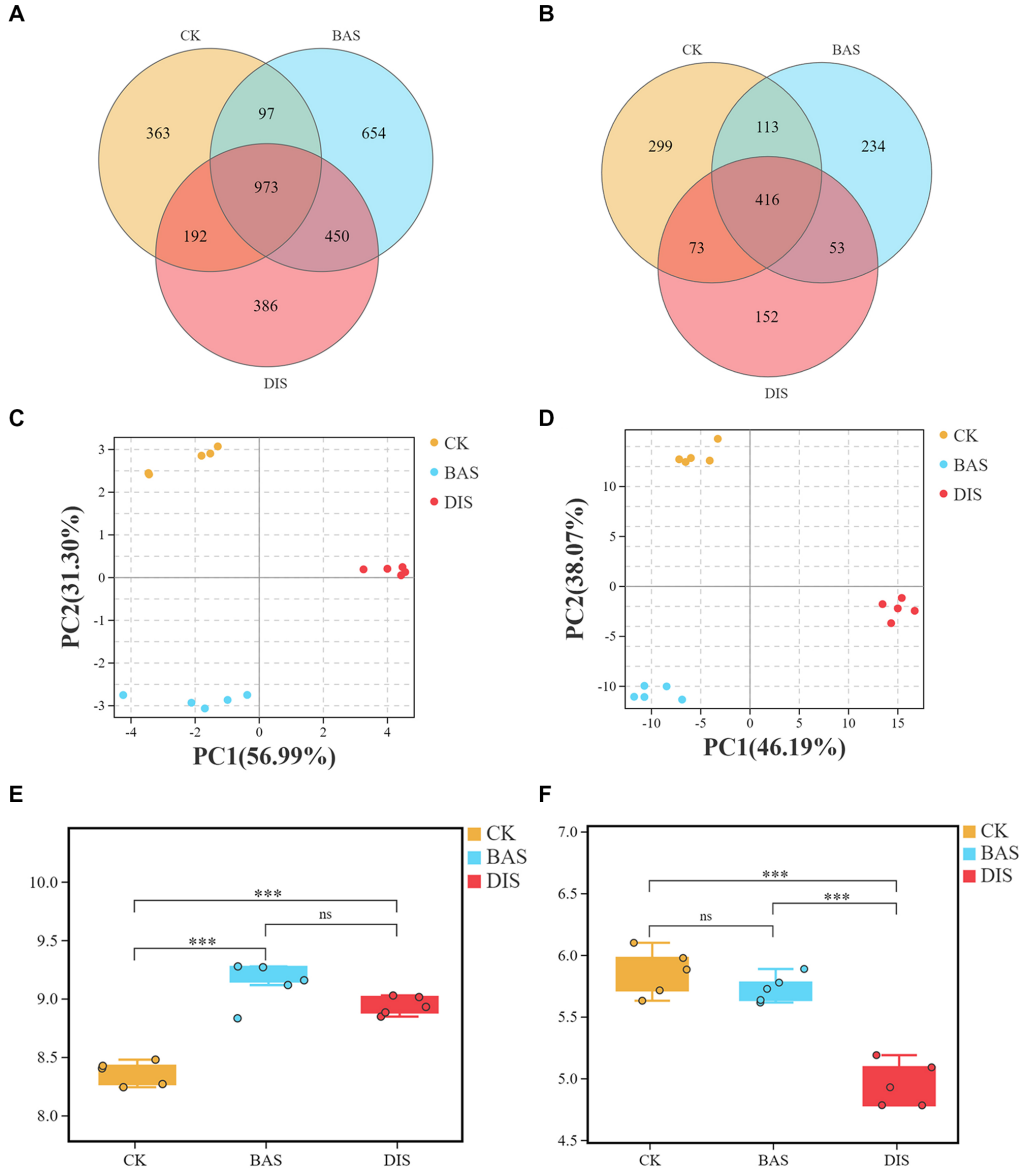
Venn analysis, PCA, and alpha diversity (Shannon index) analysis of soil bacterial and fungal diversity in the CK, BAS process, and DIS process (*n* = 5). Venn analysis of **(A)** bacterial and **(B)** fungal OTUs. PCA of **(C)** bacterial and **(D)** fungal OTUs. **(E)** and **(F)** Boxplot showing the alpha diversity (i.e., Shannon index of **(E)** bacteria and **(F)** fungi) in the CK, BAS process, and DIS process. The top and bottom whiskers of the boxes indicate the maximum and minimum values, respectively; the top margin of the box indicates the upper quartile, and the lower margin of the box indicates the lower quartile. The scattered points indicate the distribution of repeated samples within each group. “***” indicates *p* < 0.001, and “ns” indicates not significant.

The effects of the BAS process and the DIS process on soil microbial diversity were clarified via soil metabarcoding analysis. A total of 3,241,995 effective metabarcoding tags were obtained from 30 samples via sequencing of the 16S rRNA V3–V4 and ITS 1–2 regions. OTU taxonomic classification is shown in Supplementary Tables S3, S4. Alpha diversity indices including Sobs, Simpson, Chao, Ace, and good’s coverage are shown in Supplementary Tables S5, S6. OTU clustering revealed an average of 2,842 bacterial OTUs (16S rDNA) and 637 fungal OTUs (ITS) per sample (Supplementary Table S2). The Venn diagram based on the OTUs revealed that 973 and 416 bacterial and fungal OTUs were shared among all samples (Figures 2A,B). The number of unique bacterial OTUs (Figure 2A) was higher in the BAS process (654) than in the CK (363) and DIS process (368), and the number of unique fungal OTUs (Figure 2B) was significantly lower in the DIS process (152) than in the CK and BAS process (299 and 234, respectively). PCA of the abundance of bacterial and fungal OTUs in the BAS process, DIS process, and CK indicated that the composition of bacterial and fungal OTUs varied among experimental groups (Figures 2C,D). Shannon index was calculated based on the alpha diversity indices to characterize soil microbial diversity. The mean Shannon index of bacterial OTUs for the CK, BAS process, and DIS process was 8.36, 9.12, and 8.93, respectively (Figure 2E). Thus, bacterial diversity was significantly higher in the BAS process than in the CK and DIS process (*p* < 0.001), and no significant difference in bacterial diversity was observed between the DIS process and the CK. The mean Shannon index of fungal OTUs for the CK, BAS process, and DIS process was 5.72, 5.85, and 4.95, respectively (Figure 2F). Thus, fungal diversity was significantly lower in the DIS process than in the BAS process and CK (*p* < 0.001), and there was no significant difference in fungal diversity between the BAS process and the CK. In sum, we found that soil bacterial diversity was higher in the BAS process than in the DIS process and the CK, and soil fungal diversity was higher in the DIS process than in the BAS process and the CK.

### 3.3. Soil metabolome

#### 3.3.1. BAS and DIS process significantly impacted soil metabolites

**Figure 3:**
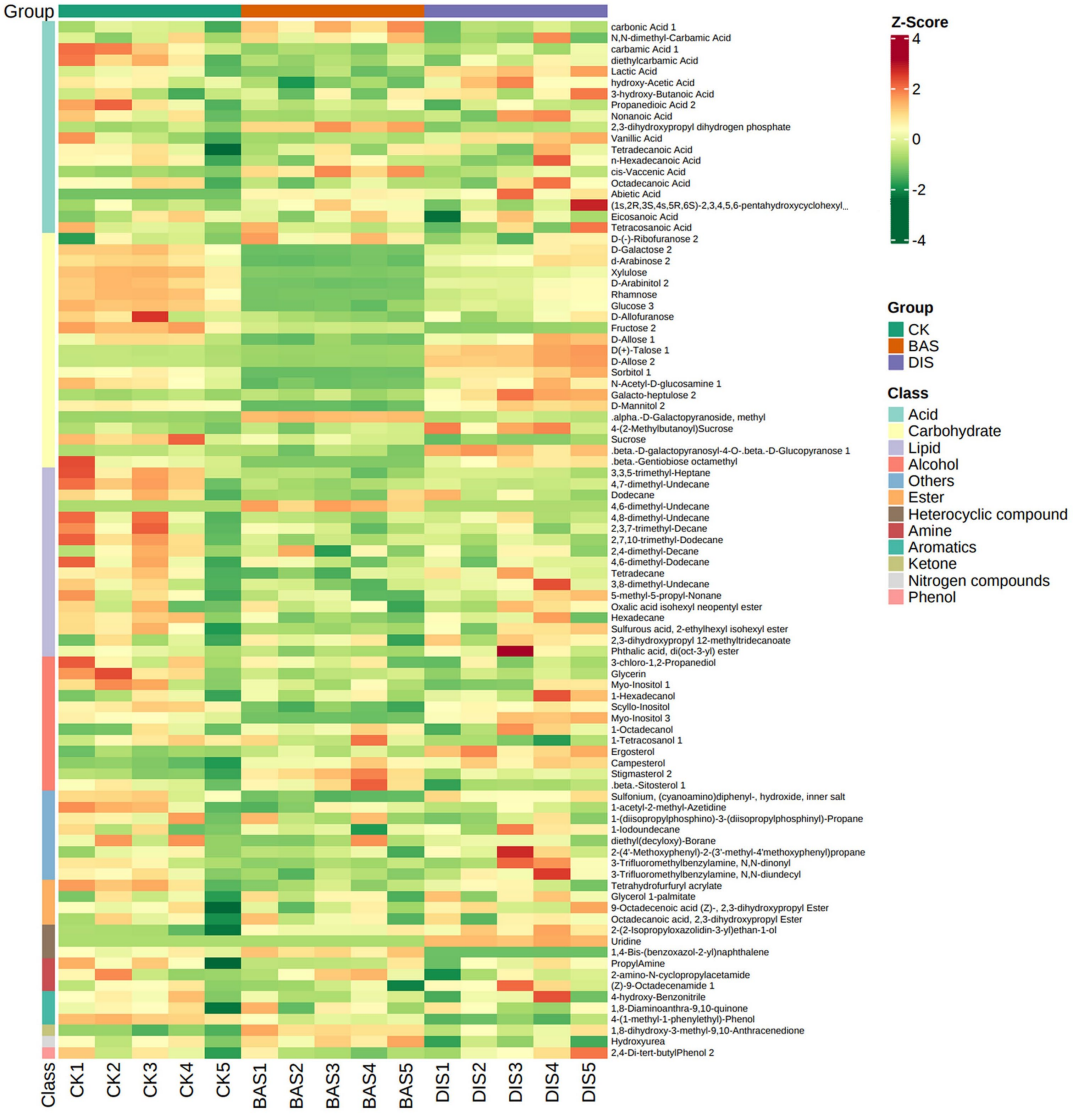
Composition of soil metabolites in the BAS process and the DIS process. Clustering heat map of all metabolites. Each column corresponds to a sample, and each row corresponds to a metabolite class. A bar with a specific color indicates the abundance of each metabolite. Shades of red and green indicate up-regulated and down-regulated metabolites, respectively.

A soil metabolomics analysis was conducted to clarify the effects of the BAS process and the DIS process on soil metabolites. A total of 96 metabolites grouped into 12 classes were detected across all soil samples, including acids (19), alcohols (12), amines (3), aromatics (3), carbohydrates (22), esters (4), heterocyclic compounds (3), ketones (1), lipids (17), nitrogen compounds (1), phenols (1), and others (8) (Figure 3; Supplementary Table S7). According to the PCA plot, the metabolites in soil in the BAS process and the DIS process significantly differed from those in the CK and were clustered into two separate groups. This suggests that both BAS and DIS processes had significant effects on the metabolites in soil (Supplementary Figure S1).

#### 3.3.2. Soil metabolites were significantly regulated in both BAS and DIS processes

The abundance of metabolites and different categories of metabolites were estimated according to *z*-scores (standardized population data). The abundances of carbohydrates were significantly affected by BAS and DIS processes according to a heat map (Figure 3). Specifically, the abundance of carbohydrates was significantly lower in the BAS process than in the CK and significantly higher in the DIS process than in the CK. Moreover, the abundance of lipids was lower in the BAS process than in the CK. Therefore, the BAS process and the DIS process had significant effects on the abundances of soil carbohydrates, and the BAS process mediated the dissolution of soil lipids.

In order to clarify the effects of the BAS process and the DIS process on soil metabolites, the top 20 most abundant metabolites were identified (Table 4). The three most abundant metabolites in the soil samples were (Z)-9-octadecenamide 1, hydroxyurea, and propanedioic acid 2. Significant variation was observed in the content of D(+)-talose, sorbitol 1, rhamnose, fructose 2, D-allose 2, and D-arabinose 2 among the experimental groups (*F*_2,12_ > 50). Specifically, the abundances of these carbohydrates were significantly lower in the BAS process than in the CK and significantly higher in the DIS process than in the CK (Table 4). The abundances of sulfonium, (cyanoamino)diphenyl-, hydroxide, inner salt; sucrose; and glycerin in soil were lower in the BAS process than in the CK, and the abundance of carbonic acid 1 was higher in the BAS process than in the CK. The abundances of hydroxyurea, sucrose, and carbonic acid were lower in the DIS process than in the CK, and the abundances of (Z)-9-octadecenamide 1; sulfonium; and (cyanoamino)diphenyl-, hydroxide, and inner salt were higher in the DIS process than in the CK. These data indicate that the BAS process and the DIS process have significant effects on the abundances of carbohydrates.

**Table 4:** The 20 most abundant soil metabolites in the BAS process and the DIS process.

Compounds	Class	*F*-value	CK/BAS	BAS/DIS
(Z)-9-Octadecenamide 1	Amine	4.302	ns	↑*
Hydroxyurea	Nitrogen compounds	8.457	ns	↓**
Propanedioic Acid 2	Acid	1.882	ns	ns
Glycerol 1-palmitate	Ester	1.068	ns	ns
n-Hexadecanoic Acid	Acid	0.124	ns	ns
Octadecanoic acid, 2,3-dihydroxypropyl Ester	Ester	0.212	ns	ns
D(+)-Talose 1	Carbohydrate	245.716	↓**	↑***
Sulfonium, (cyanoamino)diphenyl-, hydroxide, inner salt	Others	37.469	↓***	↑***
Octadecanoic Acid	Acid	1.117	ns	ns
Sucrose	Carbohydrate	19.727	↓**	↓*
Sorbitol 1	Carbohydrate	124.475	↓***	↑***
1-acetyl-2-methyl-Azetidine	Others	1.835	ns	ns
3-Trifluoromethylbenzylamine, N,N-dinonyl	Others	2.764	ns	ns
Rhamnose	Carbohydrate	56.806	↓***	↑***
2,4-Di-tert-butylPhenol 2	Phenol	1.371	ns	ns
Fructose 2	Carbohydrate	90.946	↓***	↑**
carbonic Acid 1	Acid	20.256	↑***	↓***
Glycerin	Alcohol	5.949	↓*	ns
D-Allose 2	Carbohydrate	294.132	↓**	↑***
d-Arabinose 2	Carbohydrate	63.603	↓***	↑***

Numbers are means of five soil samples; “/” indicates versus; “↑ and ↓” indicate up-regulated and down-regulated, respectively; “*” indicates *p*-value between 0.01 and 0.05; “**” indicates *p*-value between 0.001 and 0.005; “***” indicates *p* less than 0.001; “ns” indicates not significant.

### 3.4. Correlation analyses evidence the regulatory relationship between factors of soil chemistry, microbial diversity, and metabolome

#### 3.4.1. Soil enzyme activity had a highly significant positive effect on soil nutrients

**Figure 4:**
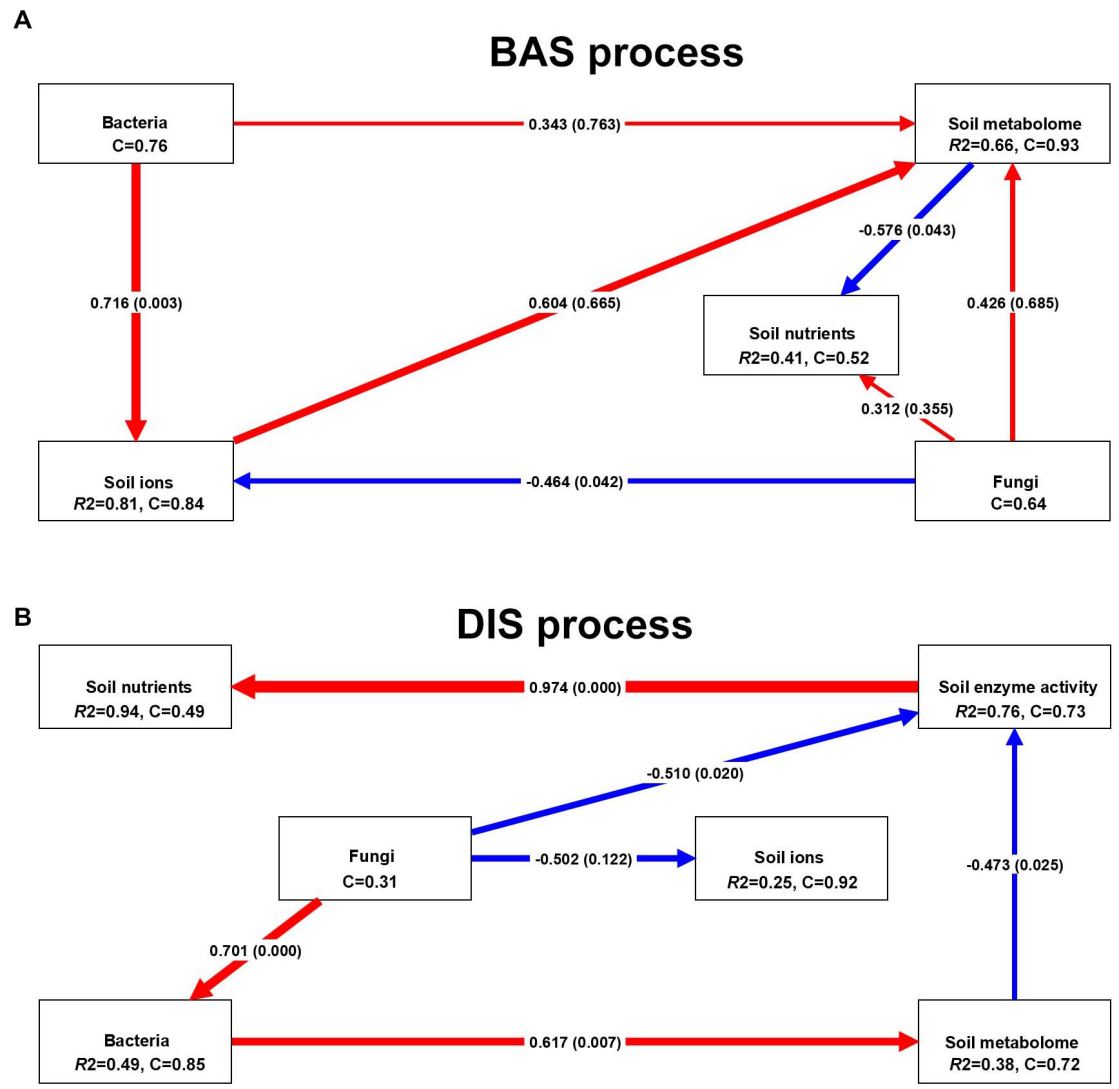
PLS–PM analysis of several soil factors, including soil enzyme activity, ions, metabolome, bacteria, and fungi, and soil nutrients in the BAS process and the DIS process. Each box corresponds to a latent variable. C indicates the Cronbach alpha index, and R2 indicates the R-squared index. The red and blue lines indicate positive and negative effects, respectively. The number in the middle of the line indicates the path coefficient, and the *p*-values are in parentheses; the thickness of the line is related to the absolute value of the path coefficient. **(A)** stands for BIS processing, and **(B)** stands for DIS processing.

The PLS–PM were built to clarify the effects of several soil factors on soil nutrients in the BAS process and the DIS process. In the BAS process (Figure 4A), the soil metabolome had a significant negative effect on soil nutrients (path coefficient = −0.576, *p* = 0.043), and fungi had a positive but insignificant effect on soil nutrients (path coefficient = 0.312, *p* = 0.335); soil ions (path coefficient = 0.604, *p* = 0.665), bacteria (path coefficient = 0.343, *p* = 0.763), and fungi (path coefficient = 0.426, *p* = 0.685) had positive effects on the soil metabolome, but none of these effects were significant (*p* < 0.05). Bacteria (path coefficient = 0.716, *p* = 0.003) and fungi (path coefficient = −0.464, *p* = 0.042) had significant positive and negative effects on soil ions, respectively. Many of the soil factors examined had significant effects on soil nutrients in the DIS process (Figure 4B). Soil enzyme activity had a highly significant positive effect on soil nutrients (path coefficient = 0.976, *p* < 0.001), which indicates that the activity of soil enzymes has a major effect on soil nutrients in the DIS process. Some soil variables affected soil nutrients through their effects on other variables; for example, fungi had a significant positive effect on bacteria (path coefficient = 0.701, *p* < 0.001); bacteria had a significant positive effect on the soil metabolome (path coefficient = 0.617, *p* = 0.007); and the soil metabolome had a negative effect on soil enzyme activity (path coefficient = −0.473, *p* = 0.025). These relationships between fungi and bacteria, between bacteria and the soil metabolome, and between the soil metabolome and soil enzyme activity drive variation in soil nutrients in the DIS process. Fungi had a negative effect on soil ions, but this effect was not significant (path coefficient = −0.502, *p* = 0.122). Our findings indicate that the BAS process had a major effect on the composition of soil ions and reveal the effects of various soil factors on soil nutrients in the DIS process.

#### 3.4.2. D(+)-talose was involved in the regulation of bacterial and fungal diversity in DIS process

**Figure 5:**
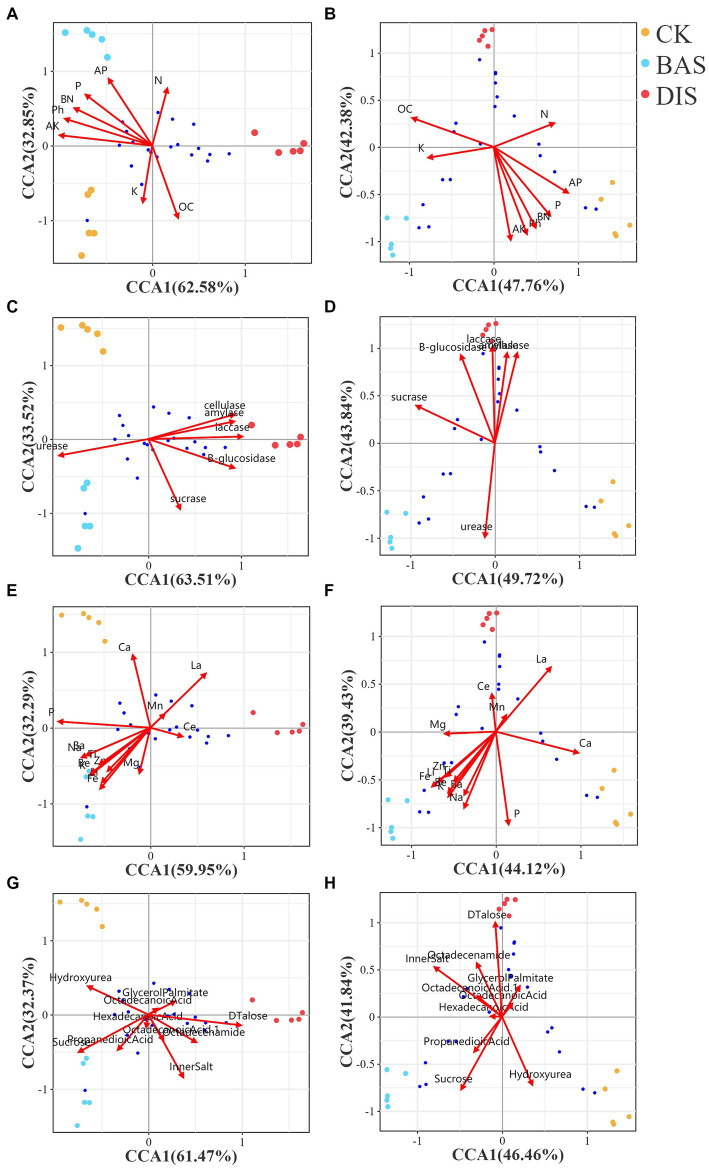
CCA with soil bacterial and fungal diversity in the BAS process and the DIS process as response variables and several factors related to soil chemistry, enzyme activity, soil ions, and the metabolome as explanatory variables. Dots of different colors indicate soil samples; the legend is located at the top right. The axes of each graph show the percentage of the total variation in OTU abundance explained. Arrows correspond to environmental factors, and the length of the arrows is positively related to the effect of environmental factors on the distribution of OTUs. Blue dots indicate OTUs; the proximity of the OTUs is positively related to their abundances in samples. CCA with soil bacterial and fungal diversity related to soil chemistry **(A** and **B)**, enzyme activity **(C** and **D)**, soil ions, **(E** and **F)** and the metabolome **(G** and **H)** were shown from top to bottom in the figure.

CCA was conducted to clarify correlations among soil chemistry, microbial diversity, and the soil metabolome in the BAS process and the DIS process. CCA was conducted using bacterial and fungal diversity based on the 50 most abundant bacterial and fungal OTUs, respectively, as response variables and other factors related to soil nutrients, enzyme activity, metal ions, and the 20 most abundant metabolites as explanatory variables (Figure 5). In the BAS process, the content of N, AP, P, and OC and the pH were related to bacterial OTU diversity (Figure 5A); however, these soil nutrient variables were not related to fungal OTU diversity in the BAS process and the DIS process (Figure 5B). The activity of cellulase, amylase, and laccase was related to bacterial OTU diversity (Figure 5C) and fungal OTU diversity (Figure 5D) in the DIS process. The content of Ba, Na, Ti, Zn, Be, Ki, Li, and Fe was related to bacterial OTU diversity in the BAS process (Figure 5E); the content of Zn, Ti, Li, Fe, Be, Ba, K, and Na was related to fungal OTU diversity in the BAS process (Figure 5F). Sucrose was related to bacterial OTU diversity in the BAS process, and D(+)-talose was related to bacterial OTU diversity (Figure 5G) and fungal OTU diversity (Figure 5H) in the DIS process. The envfit *p*-values for all the aforementioned correlations were less than 0.01.

## 4. Discussion

It was found that BAS and DIS processes had significant effects on the content of various nutrients in soil. The application of sugarcane bagasse to the fields enhanced soil nutrient conditions (Seleiman and Kheir, 2018), especially the content of N and P (Table 1). The inverse content of OC in BAS and DIS might be related to increased bacterial activity. Soil transformation may have been mediated later in DIS process. For example, Xiao et al. (2018) noted that fungal diversity can regulate soil OC mineralization, and our findings indicate that fungal diversity was lower in the BAS process than in the CK (Figure 2E), which might affect the soil mineralization process. The fact that the content of OC was higher in the DIS process than in the CK, appeared to be mediated by the cultivation of DI in light of the previous finding that fungal diversity regulates soil OC mineralization. The fact that soil pH was higher in the BAS process than in the CK (Table 1), is consistent with the results of Mohamed (Mohamed et al., 2019) showing that the BAS process can increase the pH of acidic soils. It was found that the content of P, AP, and AK was significantly higher in the BAS process than in the CK. These nutrient elements are likely derived from ground bagasse, which contains a large amount of the above elements (Sharma et al., 2015). In the DIS process, the cultivation of DI consumes a large amount of soil nutrients, especially P, AP, and AK (Table 1). A previous study of the soil nutrients in soils with DI under continuous cropping over 3 years has shown that the cultivation of DI can increase the content of soil nutrients such as N, P, and K. However, the content of AP and AK decreased gradually over the continuous cropping period (Tong et al., 2021). The content of soil nutrients decreased in the DIS process, which is inconsistent with the results of this previous study. This might be explained by differences in the design of the experiment. For example, in the previous experiment, sugarcane was not planted on the ground as in this experiment. Nevertheless, the findings in this work, as well as the results of this previous study, indicate that DI consumes large amounts of AP and AK in soil. Thus, supplementation of AP and AK is important for enhancing the productivity of the sugarcane–DI intercropping system.

Soil N is generated via the action of urease, but the activity of soil urease is often excessive, and this results in the release of excess N (Hendrickson and Douglass, 1993; Cantarella et al., 2018; Fu et al., 2020). In this study, the content of N and BN in the BAS process and the DIS process did not significantly differ, suggesting that the rate of soil N release in the BAS process and the CK exceeded the sugarcane absorption capacity. This likely caused the urease activity to decrease in the DIS process; however, little variation was observed in the content of N in the DIS process (Tables 1, 2). The above findings suggest that the DIS process inhibits soil urease activity and reduces the rate of soil N loss. Variation in the activity of sucrase and β-glucosidase was similar to variation in OC, suggesting this might be related to changes in the OC content. The significant high activity of β-glucosidase, cellulase, laccase, and amylase in the DIS process, demonstrate that these are common enzymes and are typically synthesized by edible fungi during cultivation (Cai et al., 1998; Sun et al., 2011; Shahryari et al., 2019). These enzymes can therefore promote can promote plant growth through their positive effects on the soil environment (Huang et al., 2010).

In this study, it was found that the BAS process enriched some soil mineral ions meaning that this process might replenish mineral ions in the soil. Among these ions, Ca is essential for plant growth, as it is required for the elongation of plant cells in both the shoots and roots (Burstrom, 1968). Ca can be released from sugarcane bagasse (Bento et al., 2019). The content of La has been shown to accelerate the conversion of soil N (Zhu et al., 2002), and it has no effect on plant growth at low concentrations (von Tucher and Schmidhalter, 2005; Hu et al., 2006). Thus, the BAS process enhances soil health by promoting increases in the content of Ca and La. The content of Fe, Cr, and Mo was lower in the BAS process than in the CK, and the content of P and Mo was lower in the DIS process than in the CK. Fe is an essential micronutrient in nearly all living organisms (Rout and Sahoo, 2015); thus, the loss of soil Fe caused by the BAS process might have a negative effect on sugarcane growth. Cr is a potentially toxic heavy metal that does not have any essential metabolic function in plants (Shahid et al., 2017); therefore, reductions in the content of Cr can enhance soil health. Mo is an essential element that plays a role in various biochemical processes in living organisms (Williams and Fraústo da Silva, 2002); although the content of Mo is low, it was much lower in the BAS process and the DIS process than in the CK. The same factors responsible for the decrease in the content of Mo in the BAS process and the DIS process might also explain the decrease in the P content in the BAS process and the DIS process.

Soil microbes play key roles in soil biogeochemical cycles (Basu et al., 2021). The present study found that the bacterial Shannon index and the number of unique OTUs were higher in the BAS process than in the CK and DIS process, which means that in this study BAS process regulates soil microbial diversity and thus improved soil nutrients. This is consistent with the results of previous studies (Silvia et al., 2014; Bhadha et al., 2020; Ullah et al., 2020). In addition, a previous study of fairy ring soil ecology has shown that increases in the abundance of single dominant fungi in soil decreases the alpha diversity index (Duan and Bau, 2021). Correspondingly, the present findings shown the Shannon index of fungi was lower in the DIS process than in the CK and the BAS process, and this likely stems from the abundance of DI in the soil in the DIS process, which indicated DIS process resulted in a single fungal species, i.e., *Dictyophora indusiata*, dominant in the soil, therefore regulating soil chemistry and metabolism active. Specifically, increases in the bacterial Shannon index in the BAS process were related to increases in soil nutrients, and decreases in the fungal Shannon index in the DIS process were related to decreases in soil nutrients. In light of a previous study suggesting that soil microbes can mediate the transformation of the aforementioned soil nutrients (Basu et al., 2021), we suggest that this finding might stem from the fact that the BAS process increases both bacterial alpha diversity and the absolute abundance of bacteria, whereas the DIS process might inhibit bacterial absolute abundance because of the growth of DI in soil. Additional quantitative microbial surveys are needed to verify this hypothesis.

Soil metabolomics can be used to clarify correlations of metabolic fingerprints with environmental factors, soil nutrients, microbial diversity, and plant phenotypes (Withers et al., 2020). It was found that a large amount of carbohydrate metabolites were consumed in the BAS process, and the consumption of carbohydrate metabolites was reduced in the DIS process. Carbohydrates are a significant component of the organic matter in all soils and commonly account for 5 to 20% of soil organic matter (Lowe, 1978); carbohydrates are thus often used as indicators of the physical quality of soil (Zaher et al., 2020). A previous study of the effects of fairy ring fungi on soil health has shown that increases in carbohydrates in soil are associated with the enhanced growth of Leymus chinensis (Duan et al., 2022); therefore, our findings indicate that the nutrient status of soils in the DIS process was superior to that in the BAS process. In addition, the content of carbohydrates was negatively correlated with the content of P, AP, and BN and positively correlated with the content of OC. Therefore, the consumption of soil OC in the BAS process might increase the content of P, AP, and BN through the consumption of soil carbohydrates, and this might be achieved via increases in soil bacterial diversity in the BAS process in light of a previous study suggesting that soil microbes play a key role in soil biogeochemical cycling and thus regulate the availability of soil mineral nutrients (Basu et al., 2021).

The identity of the most abundant soil metabolites was consistent with the above results. Carbohydrates were not the only important class of metabolites affected by the BAS process and the DIS process, but they were some of the most abundant metabolites. D(+)-talose 1 was the most abundant carbohydrate metabolite, and variation in its abundance was consistent with the effects of the BAS process and the DIS process on carbohydrate metabolites (down-regulated in the BAS process and up-regulated in the DIS process). A previous study has shown that the content of D-talose is affected by root rot disease; this suggests that D-talose could be used as an indicator of soil health.

PLS–PM and CCA showed that the inhibition of urease in the DIS process was the main factor affecting soil nutrients, as soil enzyme activity is the main regulatory factor of soil nutrients and then the activity of urease was higher than that of all soil enzymes combined, which suggest cultivation of *Dictyophora indusiata* in sugarcane field effectively could improve soil health. Given that the activity of urease mainly affects the soil N cycle (Fu et al., 2020), other factors might jointly regulate the content of other nutrients, such as P and AK. This might be related to the effect of the DIS process on the abundance of soil bacteria, given that increases in bacterial diversity were associated with increases in soil nutrients. According to PLS–PM (Figure 4B), enzyme activity affected soil nutrients, and this was achieved via the effects of bacteria on the soil metabolome, which were mediated by the effects of fungi on bacteria. D(+)-talose 1 was correlated with bacterial and fungal diversity in the DIS process according to CCA (Figures 5G,H), indicating that it is a key biomarker and was affected by the DIS process. The DIS process inhibits the activity of urease and thus reduces the rate of soil N loss. Our findings thus suggest that D(+)-talose 1 might be related to the regulation of soil urease. Future studies of soil fertility are needed to clarify whether D(+)-talose 1 inhibits urease activity.

## 5. Conclusion

The present study evaluated the effects of a sugarcane–DI intercropping system and bagasse application on soil health. The findings indicate that the sugarcane–DI system can promote soil health. This study found that this intercropping system could reduce soil N loss, and also explored potential mechanisms underlying these observations, and these findings have implications for the development of novel soil urease inhibitors. In future studies, we plan to characterize the effects of DI on the abundances of soil microbes and the relationship between soil urease activity and the metabolite D(+)-talose 1.

## Data availability statement

The datasets presented in this study can be found in online repositories. The names of the repository/repositories and accession number(s) can be found at: https://www.ncbi.nlm.nih.gov/genbank/, PRJNA924520.

## Author contributions

MD, ZW, and TH contributed to the conception and design of the study. MD, YL, GZ, SL, XL, XW, HH, JQ, SQ, BF, Q-HL, CL, QL, and LW performed investigation. MD and YL performed the statistical analysis. MD wrote the first draft of the manuscript. All authors contributed to the article and approved the submitted version.

## Funding

This work was jointly funded by the National Natural Science Foundation of China [grant number NNSFC 32260715], Central Government Guides Local Funds for Science and Technology Development [grant number GuiKe ZY21195033], Guangxi Major Science and Technology Project [grant number GuiKe AA22117004], Guangxi Science and Technology Base and Special Talent [grant number GuiKe AD20297130], Science and Technology Pioneer Special of Guangxi Academy of Agricultural Sciences [grant number GuiNongKeMeng 202203-1-2], and Team Project for Guangxi Academy of Agriculture Sciences [grant number Guinongke 2021YT004].

## Conflict of interest

The authors declare that the research was conducted in the absence of any commercial or financial relationships that could be construed as a potential conflict of interest.

## Publisher’s note

All claims expressed in this article are solely those of the authors and do not necessarily represent those of their affiliated organizations, or those of the publisher, the editors and the reviewers. Any product that may be evaluated in this article, or claim that may be made by its manufacturer, is not guaranteed or endorsed by the publisher.

## Footnotes

^1^ https://www.smartpls.com/
