# Supplementary figures and images for: Soil chemistry, metabarcoding, and metabolome analyses reveal that a sugarcane—Dictyophora indusiata intercropping system can enhance soil health by reducing soil nitrogen loss
Source: Front Microbiol. 2023 May 25;14:1193990. doi: 10.3389/fmicb.2023.1193990 (PMC10249477; doi:10.3389/fmicb.2023.1193990)

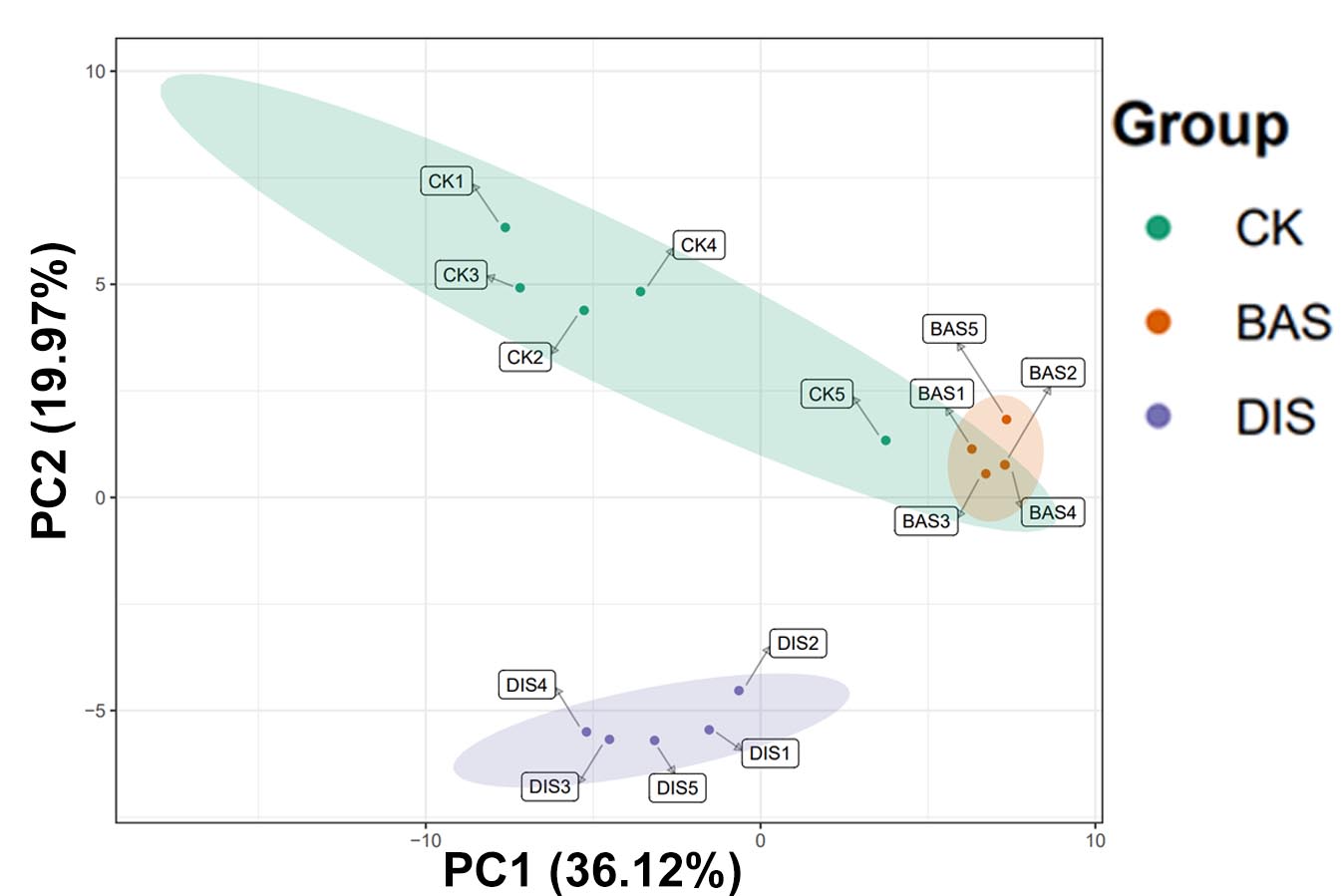

Supplement: Supplementary file 8 [file Image_1.JPEG]
